# Antipyretic and antibacterial activity of *Chloranthus erectus* (Buch.-Ham.) Verdcourt leaf extract: A popular folk medicine of Arunachal Pradesh

**DOI:** 10.4103/0253-7613.70083

**Published:** 2010-10

**Authors:** Hui Tag, Nima D. Namsa, M. Mandal, P. Kalita, A.K. Das, S.C. Mandal

**Affiliations:** Pharmacognosy Research Laboratory, Department of Botany, Rajiv Gandhi University, Rono Hills, Itanagar - 7911 13, Arunachal Pradesh, India; 1 Department of Microbiology and Cell Biology, Indian Institute of Science, Bangalore - 560 012 India; 2 Department of Molecular Biology and Biotechnology, Tezpur University, Tezpur - 784 028, Assam India; 3 Division of Pharmacognosy, Department of Pharmaceutical Technology, Jadavpur University, Jadavpur, Kolkata - 700032, India

**Keywords:** Antipyretic activity, *Chloranthus erectus*, *Khampti* tribe

## Abstract

**Objective:**

The main objective of this work was to study the antipyretic and antibacterial activity of *C. erectus* (Buch.-Ham.) Verdcourt leaf extract in an experimental albino rat model.

**Materials and Methods:**

The methanol extract of *C. erectus* leaf (MECEL) was evaluated for its antipyretic potential on normal body temperature and Brewer’s yeast-induced pyrexia in albino rat’s model. While the antibacterial activity of MECEL against five Gram (−) and three Gram (+) bacterial strains and antimycotic activity was investigated against four fungi using agar disk diffusion and microdilution methods.

**Result:**

Yeast suspension (10 mL/kg b.w.) elevated rectal temperature after 19 h of subcutaneous injection. Oral administration of MECEL at 100 and 200 mg/kg b.w. showed significant reduction of normal rectal body temperature and yeast-provoked elevated temperature (38.8 ± 0.2 and 37.6 ± 0.4, respectively, at 2–3 h) in a dose-dependent manner, and the effect was comparable to that of the standard antipyretic drug–paracetamol (150 mg/kg b.w.). MECEL at 2 mg/disk showed broad spectrum of growth inhibition activity against both groups of bacteria. However, MECEL was not effective against the yeast strains tested in this study.

**Conclusion:**

This study revealed that the methanol extract of *C. erectus* exhibited significant antipyretic activity in the tested models and antibacterial activity as well, and may provide the scientific rationale for its popular use as antipyretic agent in *Khamptis’s* folk medicines.

## Introduction

*Chloranthus erectus* (Buch.-Ham.) Verdcourt (Chloranthaceae) is a shrub distributed in shade habitat, near stream, and forest floor of tropical and temperate zone of Eastern Himalayan, Indo-Burma, and South East Asian region.[1,2] *C. erectus* is one such plant listed in *“materia medica”* of *Khampti’s* tribe written in Thai scripts with mentions of about 10–15 plants used in single herbal formulation to cure the common ailments of their locality.[3,4] *C. erectus* is a popular folk medicine traditionally used by the *Khampti* tribe of Aruanachal Pradesh, India for curing localised swelling, joint pain, skin inflammation, wound healing, fever, and body ache. The *Khampti* tribes are good in herbal medicines and have a rich reservoir of plant traditional knowledge.[5] However, few scientific reports are available with respect to their pharmacological and phytochemical properties.[6–8] In our earlier study, the methanol extract of *C. erectus* leaf (MECEL) has shown significant anti-inflammatory activity.[9] On the basis of the traditional use of the plant as an antipyretic and wound healing agent, we have evaluated the MECEL for possible antipyretic activity in an experimental albino rat model and antibacterial activity by agar-disk diffusion method to substantiate the folklore claim. The effect of the methanol extract was also compared with that of the standard drug—paracetamol, a well-known antipyretic agent.

## Materials and Methods

### Plant material

The leaves of *C. erectus* (Buch.-Ham.) Verdcourt were collected from the Namsai forest of Lohit district, Arunachal Pradesh, India. The plant material was taxonomically identified and authenticated by Dr. A.A. Mao, Botanical Survey of India, Shillong. A voucher specimen (number Hui 041HAU:2003) was deposited in the herbarium of the Department of Botany, Rajiv Gandhi University for our future reference. The leaves were dried under shade, powdered (1 kg) and passed through 40-mesh sieve, and stored for further use.

### Preparation of methanolic extract

The powdered leaves were extracted using methanol in a Soxhlet extraction apparatus. One kilogram of powdered plants material was extracted in cold with 90% methanol a as solvent for 72 h at room temperature. The solvent was then evaporated to dryness under reduced pressure in rotary evaporator at 45–70°C. The concentrated methanol extracts of MECEL (yield of ± 110 g, 11.1% w/w) was stored in a desiccator for future use. The chemical constituents of the MECEL such as tannins, saponins, flavanoids, steroids, terpenoids, gums, reducing sugars, alkaloids, and anthroquinone were identified by qualitative analysis[9] as described previously.

### Animals used: antipyretic activity test

Healthy albino rats (Wistar strain) of either sex weighing 180–200 g were used, and all the animal experiments were carried out according to the institutional regulations and national criteria for animal experimentation[9] as described previously. The experimental data were statistically evaluated using one-way analysis of variance (ANOVA) (SYSTAT version 10.0). Paired statistical analyses were performed using the Student’s *t*-test and the *P*-values were considered statistically significant at *P* < 0.05 and *P* < 0.001.

### Study on normal body temperature of rats

Animals were divided into four groups comprising six rats in each group. Only healthy rats with constant normal body temperature were selected for this experiment. The body temperature of each rat was measured rectally at predetermined intervals before and for 5 h after administration of either 2% aqueous Tween-80 solutions (vehicle control) or MECEL at doses of 100 and 200 mg/kg orally.

### Yeast-induced hyperthermia in rats

Rats of either sex were divided into four groups, comprising six rats in each group. The normal body temperature of each rat was measured with digital thermometer rectally at predetermined intervals and recorded. The rats were trained to remain quiet in a restraint cage. Fever was induced by a subcutaneous injection of 10 mL/kg of 15% (w/v) yeast suspended in 0.5% (w/v) methylcellulose solution.[10] Rats were then returned to their housing cages. After 19 h of yeast injection, the third and fourth group of animals received MECEL orally at doses of 100 and 200 mg/kg, respectively. The first group received 5 mL/kg body weight of 2% (v/v) aqueous Tween-80 solution orally (vehicle control group). The second group received standard drug paracetamol (150 mg/kg). Temperature was recorded at 1 h intervals up to 23 h after yeast injection.

### Antimicrobial assay

The *in vitro* antibacterial and antifungal assays were carried out by adopting the modified agar-disk diffusion method. For the antimicrobial assay, the dried extracts were dissolved in 1% dimethylsulfoxide (DMSO) to a final concentration of 100 mg/mL and sterilized by filtration through a 0.45-*μ*m membrane filter. Mueller-Hinton Agar was inoculated with (overnight, 12 h) bacterial cell suspension (200 *μ*L in 20 mL medium, 5 × 10^5^ CFU/mL). Sterile filter paper disks of 6-mm diameter were impregnated with 20 *μ*L extracts (equivalent to 2 mg of MECEL/disk) and after complete evaporation, the disks were placed on the surface of the inoculated agar plates. Chloramphenicol and nystatin were used as reference antibiotics against bacteria and yeast, respectively. Negative controls were done using paper discs loaded with 20 *μ*L of the solvent (DMSO). The plates were incubated at 37°C for 18 h. Similarly, Sabouraud Dextrose Agar was inoculated with yeast overnight cell suspension and incubated at 28°C for 48 h. At the end of the incubation period, the antimicrobial activities were evaluated by measuring the zone of inhibition. An inhibition zone of 15 mm or greater (including diameter of the disk) was considered as high antimicrobial activity in this study and evaluated their minimum inhibitory concentration (MIC) following the protocol as described previously.[11]

### Microorganisms used

The following microorganisms were used for *in vitro* microbial sensitivity investigation: *Staphylococcus aureus* MTCC 96; *Bacillus subtilis* MTCC 736, *B. subtilis* DW1 strain, *Escherichia coli* MTCC 739, *Pseudomonas aeruginosa* ATCC 25619, *Salmonella typhimurium* ATCC 6539, *Ralstonia eutropha* MTCC 98; *Klebsiella pneumonia* (Defence Research Laboratory, Solmara, Tezpur, Assam), *Candida albicans* (School of Tropical Medicines, Kolkata); Arunachal Pradesh strain *Candida glabrata* MTCC 3985, Manipur strain *Debangomyces hansenii* MTCC 3977, and *Saccharomyces cerevisiae* MTCC 3980.

## Results

### Effects of MECEL on normal and yeast-induced hyperthermia

It was observed that the MECEL at 100 mg/kg caused lowering in normal rectal body temperature up to 4 h (*P* < 0.01) following its administration. This effect was maximal at 4 h and MECEL at doses of 200 mg/kg caused a significant lowering of rectal temperature of rats from 36.4 ± 0.2°C to 35.4 ± 0.21°C up to 5 h after extract administration [Table 1]. It was found that the subcutaneous injection of yeast suspension markedly elevated the rectal temperature of rats following 19 h of its administration. MECEL at doses of 100 and 200 mg/kg caused significant reduction of rectal temperature of hyperthermic rats by 38.8 ± 0.2 and 37.6 ± 0.4, respectively, in a dose-dependent manner [Table 2]. The antipyretic effect started as early as 2 h and the effect was maintained for 4 h following its administration. The standard drug paracetamol (150 mg/kg) significantly reduced the yeast-induced elevation of body temperature. The results obtained from both MECEL-treated and standard drug—paracetamol-treated rats were compared with vehicle control (2% aqueous Tween-80 solution (v/v) and we observed a significant reduction in the yeast-elevated rectal temperature in rats [Table 2]. The antipyretic effects of MECEL on normal body temperature and hyperthermic rats were dose-dependent [Tables 1 and 2].

**Table 1:** Antipyretic activity of MECEL on normal rat body temperature

*Treatment (mg/kg)*	*Rectal temperature (°C) before and after treatment*					
	*0 h*	*1 h*	*2 h*	*3 h*	*4 h*	*5 h*
Control (5 mL/kg)	36.5 ± 0.3	36.6 ± 0.3	36.8 ± 0.12	36.7 ± 0.6	36.7 ± 0.3	36.4 ± 0.2
Paracetamol, 150	36.3 ± 0.2	36.1 ± 0.6	35.9 ± 0.23	35.8 ± 0.3	36.1 ± 0.13*	36.5 ± 0.1*
MECEL, 100	36.4 ± 0.1	35.2 ± 0.4**	35.2 ± 0.18**	35.3 ± 0.2**	35.8 ± 0.21**	35.9 ± 0.4**
MECEL, 200	36.1 ± 0.5	33.5 ± 0.2*	35.0 ± 0.22*	35.01 ± 0.1*	35.1 ± 0.3*	35.4 ± 0.21**

Data represent mean ± SE (standard error) (*n* = 6)

* *P* < 0.001

** *P* < 0.01, significant from the control; control 2% (v/v) aqueous Tween-80 solution; MECEL, methanol extract of *C. erectus* leaf.

**Table 2:** Antipyretic activity of MECEL on yeast-induced hyperthermia test in rats

*Treatment (mg/kg)*	*Rectal temperature (°C)*					
	*Before yeast*	*19 h after yeast*	*Time after MECEL treatment (min)*			
			*60*	*120*	*18*	*240*
Control (5 ml/kg)	37.14 ± 0.1	38.6 ± 0.11	38.3 ± 0.31	38.4 ± 0.17	38.5 ± 0.21	38.6 ± 0.12
Paracetamol, 150	37.2 ± 0.2	39.2 ± 0.41	37.6 ± 0.16*	37.5 ± 0.3*	37.1 ± 0.11*	37.0 ± 0.2*
MECEL, 100	37.55 ± 0.4	39.12 ± 0.32	38.8 ± 0.2**	38.21 ± 0.15**	38.2 ± 0.14*	38.1 ± 0.16*
MECEL, 200	37.18 ± 0.2	39.5 ± 0.31	37.6 ± 0.4**	37.4 ± 0.12*	37.3 ± 0.24*	37.1 ± 0.3*

Data represent mean ± SE (standard error) (*n* = 6)

* *P* < 0.001

** *P* < 0.01, significant from the control; control 2% (v/v) aqueous Tween-80 solution; MECEL, methanol extract of *C. erectus* leaf.

### Antibacterial activity of MECEL

MECEL at 2 mg/disk showed broad spectrum of growth inhibition activity against both G (−) and G (+) bacterial strains tested in this study [Table 3]. However, MECEL was not effective against the yeast strains tested in this study. MIC values of MECEL obtained against few selected bacteria included *S. aureus* (800 *μ*g/mL), *B. subtilis* (600 *μ*g/mL), *E. coli* (650 *μ*g/mL), and *P. aeruginosa* (900 *μ*g/mL).

**Table 3:** Antibacterial activity of MECEL against various microorganisms using agar-disk diffusion method (2 mg/disk)

*Test organisms*	*Zone of inhibition (mm)^a^*	*MIC (μg/ml)^b^*	*Standard antibiotic^c^*
*Staphylococcus aureus*	16.0 ± 0.10	800.0 ± 0.12	20
*Bacillus subtilis*	18.0 ± 0.25	600.0 ± 0.25	22
*B. subtilis* DW1 strain	16.0 ± 0.20	850.0 ± 0.00	22
*Escherichia coli*	19.0 ± 0.40	650.0 ± 0.12	25
*Klebsiella pneumonia*	14.0 ± 0.12	ND	20
*Pseudomonas aeruginosa*	15.0 ± 0.00	900.0 ± 0.25	22
*Ralstonia eutropha*	14.0 ± 0.25	ND	24
*Salmonella typhimurium*	14.0 ± 0.00	ND	21
*Candida albicans*	×	×	20
*Saccharomyces cerevisiae*	×	×	22
Candida glabrata	×	×	24
Debangomyces hansenii	×	×	23

a The values for zone of growth inhibition including the diameter of the paper disk (6 mm) are presented as mean ± SD;

b minimal inhibitory concentration (MIC) was determined using microdilution technique;

c chloramphenicol (10 μg/mL) and nystatin (10 μg/mL) as standard control for bacteria and yeast, respectively; “x”: indicate no activity; ND, not determined; MECEL, methanol extract of *C. erectus* leaf.

## Discussion

An inflammatory response has been associated with various manifestations such as elevated body temperature and pain. Hence, a drug having anti-inflammatory activity may also show antipyretic and antibacterial properties. Preliminary pharmacological screening experiments were conducted (data not shown) with crude *C. erectus* leaf extracts (methanol, acetone, hexane, chloroform, ethyl acetate, and water), only the methanol fractions were found to exhibit significant antipyretic activity whose effect is comparable to that of standard drug—paracetamol reported in this study. Among the various organic solvents tested, the methanol fraction may facilitate the solubility of a mixture of active phytocomponents due to its high polarity. Previously, we have shown that the MECEL has significant anti-inflammatory property[9] in an albino rat model. This study showed that the MECEL possesses a significant antipyretic property in an experimental brewer’s yeast-induced hyperthermia in rats. Two different doses of MECEL (100 and 200 mg/kg) were administered orally 19 h post subcutaneous injection of brewer’s yeast. The rats treated with MECEL showed a significant reduction in rectal temperature in a dose-dependent manner as early as 2 h post hyperthermia-induction (i.e., from 38.8 ± 0.31°C to 37.6 ± 0.4°C, 200 mg/kg, Table 2, *P* < 0.01) when compared with control animals (vehicle treated) versus treated with paracetamol (37.6 ± 0.16°C at 150 mg/kg, Table 2). This study reveals that the MECEL causes a significant antipyretic effect in yeast-provoked elevation of body temperature [Table 2] as well as normal body temperature in rats [Table 1]. Under both experimental conditions, the MECEL caused a significant lowering of body temperature whose antipyretic effect is comparable to that of paracetamol. Fever may be a result of infection, inflammation, or other disease states. The body temperature requires a delicate balance between the production and loss of heat and is regulated by hypothalamus.[12] Suppression of elevating body temperature might be due to the combination of anti-inflammatory and antipyretic effects of the MECEL. These results suggest that the plant probably has some influence on prostaglandins (PGs) biosynthesis because PGs[13] are believed to be regulators of body temperature. However, this effect needs further study to ascertain the exact mechanism of action.

MECEL exhibited a broad spectrum of growth inhibition activity against both G (−) and G (+) bacterial strains and could be due to the presence of secondary metabolites.[9] The ineffectiveness of MECEL against fungi may be related with inability of the methanol soluble compounds to penetrate the hydrophobic and hydrophilic lipid bilayers architecture of yeast cell wall.[14] The antipyretic effects of MECEL in yeast-provoked elevation of body temperature as well as normal body temperature in rats will be studied further to understand the exact molecular basis for the observed antipyretic property. The results obtained in this study probably support the traditional application of *C. erectus* in the treatment of fever and bacterial infections.
